# Design and Development of Modified mRNA Encoding Core Antigen of Hepatitis C Virus: a Possible Application in Vaccine Production

**DOI:** 10.29252/.23.1.57

**Published:** 2019-01

**Authors:** Zarin Sharifnia, Mojgan Bandehpour, Bahram Kazemi, Nosratollah Zarghami

**Affiliations:** 1 Drug Applied Research Center, Tabriz University of Medical Sciences, Tabriz, Iran; 2 Department of Medical Biotechnology, Faculty of Advanced Medical Sciences, Tabriz University of Medical Sciences, Tabriz, Iran; 3 Cellular and Molecular Biology Research Center, Shahid Beheshti University of Medical Sciences, Tehran, Iran; 4 Department of Biotechnology, School of Advanced Technologies in Medicine, Shahid Beheshti University of Medical Sciences, Tehran, Iran; 5 Department of Clinical Biochemistry and Laboratories Medicine, Faculty of Medicine, Tabriz University of Medical Sciences, Tabriz, Iran

**Keywords:** Hepatitis C, Messenger, RNA, Vaccines

## Abstract

**Background:**

Hepatitis C virus (HCV) is a blood-borne pathogen, resulting in liver cirrhosis and liver cancer. Despite of many efforts in development of treatments for HCV, no vaccine has been licensed yet. The purpose of this study was to design and prepare a specific mRNA, without 5’ cap and poly (A) tail transcribed *in vitro* capable of coding core protein and also to determine its functionality.

**Methods:**

Candidate mRNA was prepared by *in vitro* transcription of the designed construct consisting of 5’ and 3’ untranslated regions of heat shock proteins 70 (hsp70) mRNA, T7 promoter, internal ribosome entry site (IRES) sequences of eIF4G related to human dendritic cells (DCs), and the *Core* gene of HCV. To design the modified mRNA, the 5’ cap and poly (A) tail structures were not considered. DCs were transfected by *in vitro-*transcribed messenger RNA (IVT-mRNA) and the expressions of green fluorescent protein (*GFP)*, and *Core* genes were determined by microscopic examination and Western blotting assay.

**Results:**

Cell transfection results showed that despite the absence of 5’ cap and poly (A) tail, the structure of the mRNA was stable. Moreover, the successful expressions of *GFP* and *Core* genes were achieved.

**Conclusion:**

Our findings indicated the effectiveness of a designed IVT-mRNA harboring the *Core* gene of HCV in transfecting and expressing the antigens in DCs. Considering the simple and efficient protocol for the preparation of this IVT-mRNA and its effectiveness in expressing the gene that it carries, this IVT-mRNA could be suitable for development of an RNA vaccine against HCV.

## INTRODUCTION

Hepatitis C virus (HCV) is a blood-borne pathogen and an enveloped, single-stranded, and positive-sense RNA virus belonging to Flaviviridae family[1,2]. It is estimated that about 2%- 3% of the world’s population is infected with HCV[3], and most of the acute hepatitis C infections become chronic. If left untreated, the chronic disease can lead to cirrhosis and hepatocellular carcinoma in a number of patients. At the moment, chronic hepatitis C infection can be treated by antiviral therapy[1,4-6]. In recent years, direct-acting antivirals (DAAs) regimens has been introduced due to its high efficacy rate (>95%) against all genotypes of the HCV. However, there are major barriers to widespread use of DAAs regimens, including geographical factors with limited availability of new compounds, virus factors like HCV genotype, host factors such as prolonged liver damage even after treatment, inability to prevent re-infection, and the high cost of DAAs drugs[7-9]. Although antiviral agents show a great efficacy in HCV treatment, it seems that the global burden of liver disease does not decrease without the combination of effective antiviral treatments, appropriate screening techniques, and preventive vaccine development. Currently, no vaccine has been licensed or administered for HCV, but trials are under way[10].

A wide variety of vaccines have been designed so far such as protein-based vaccines like recombinant glycoproteinE1/glycoprotein E2 adjuvanted with MF59 and recombinant HCV core antigen formulated with the T-cell adjuvant IMX, inactivated cell culture-derived HCV virions, virus-like particles presenting HCV envelope proteins, viral-vectored vaccines expressing conserved HCV NS3-NS5B non-structural proteins (Ad6-Nsmut/AdCh3-Nsmut and MVA-NSmut/AdCh3-NSmut vaccines), as well as DNA plasmid encoding the HCV proteins 3/4a[11,12].

mRNA is a transient carrier of genetic information that is naturally metabolized and does not integrate to the genome. The synthetic mRNA can be easily designed to express any antigen with high efficiency, and it can also act as an adjuvant in vaccine formulations. Since mRNA, in contrast to DNA, is located and acts in the cytoplasm, it easily can transfect all cell types, especially hard-to-transfect cells. Another advantages of using mRNA in a treatment approach are its cost-effective manufacturing and its easy scalability. As mRNA transcription is possible *in vitro*, production of high dose vaccines in a short time is achievable, which allows the rapid production of a vaccine for a new antigen during pandemics[13-17]. Typically, RNA vaccines consist of a mRNA synthesized via an *in vitro* transcription using a bacteriophage RNA polymerase and a DNA template encoding the antigen of interest [18].

Vaccine development using mRNA approaches have advantages over other vaccine methods[19]. mRNA vaccines is promising to prevent and treat a wide range of diseases such as influenza, rabies, or cancers[18,20]. However, no HCV vaccine has been designed using this methodology.

Recently, studies have used *in vitro* transcribed- messenger RNA (IVT-mRNA), as a gene carrier, in subunit vaccines to supply therapeutic proteins. *in vitro* protein expression of brome mosaic virus and poliovirus RNA has been confirmed[21,22], and based on this fact, several vaccines have been designed against viral infections[23]. Studies have indicated the effectiveness of dendritic cells (DCs) transfected with antigen-encoding mRNA genes in cancer immunotherapy[24,25]. Although mRNA approaches is being used widely in several clinical trials in cancer therapy[26], IVT-mRNA is supposed to be too immunogenic and labile for genetic introduction of proteins. To overcome these challenges, several strategies have been developed, that include the replacement of modified nucleotides with unmodified counterpart, codon optimization of an IVT-mRNA sequence for enhance protein production, using untranslated regions (UTRs) in the structure of mRNA, and the use of safe delivery tools[27]. In order to develop the cheaper and more flexible technology of IVT-mRNA production, focusing on the above mentioned strategies may be useful.

Our aim here was to design and prepare a synthetic mRNA that en codes the most conserved protein of HCV, core protein and also to determine its ability to encode the protein in DCs. In the present study, we have made a sequence consisted of 5’ and 3’ UTRs of heat shock proteins 70 (hsp70) mRNA, T7 promoter, internal ribosome entry site (IRES) fragments that enable eIF4G capture for the initiation of translation to express HCV *Core* gene and Core/signal peptide (SP) in human DCs.

## MATERIALS AND METHODS

### Plasmid constructs

**Fig. 1:**
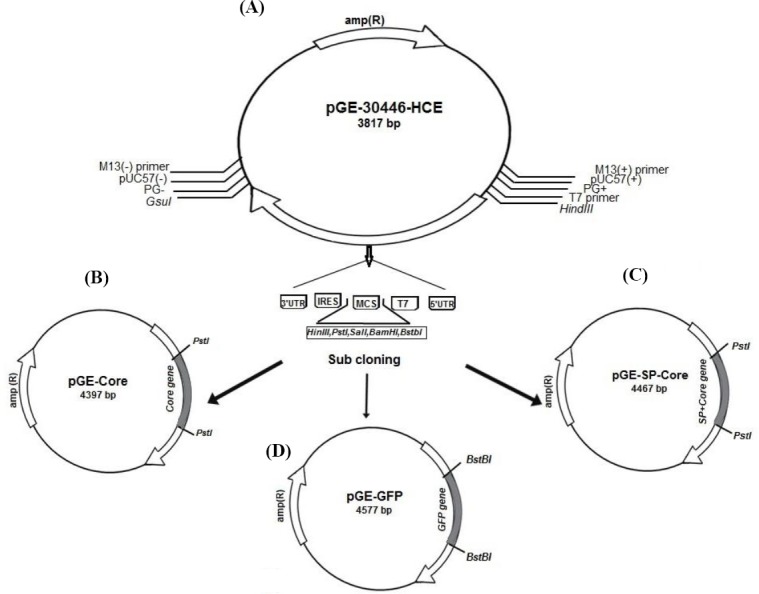
Schematic representation of plasmid construction procedure. (A) The designed pGE-30446-HCE plasmid containing synthesis gene with the following characteristics: gene name, 30446- HCE; gene length, 915 bp; orientation, M13forward-gene-M13reverse; cloning site, Ec072I; vector, pGE; (B) pGE-30446-HCE plasmid after subcloning of HCV Core protein-coding sequence in *Pst*I restriction site; (C) pGE-30446-HCE plasmid after subcloning of HCV SP-Core-coding sequence in *Pst*I restriction site; (D) pGE-30446-HCE plasmid after subcloning of GFP-coding sequence in *Bst*BI restriction site.

To prepare a pGE-Core construct, Core protein consisting of 1–191 aa (573 bp) of HCV-1a strain Tehran 12 (GenBank: AF512996.1, 2002) was ligated into the pGE-30446-HCE vector using *Pst*I restriction enzyme. This plasmid was constructed under the control of T7 bacteriophage promoter with 5’ UTR, ORF, IRES, and 3’ UTR. The 5’ UTR and 3’ UTR were from human hsp70 along with IRES sequences of human DCs This synthetic construct was provided commercially(Generay, China (Fig. 1A).

**Fig. 2:**
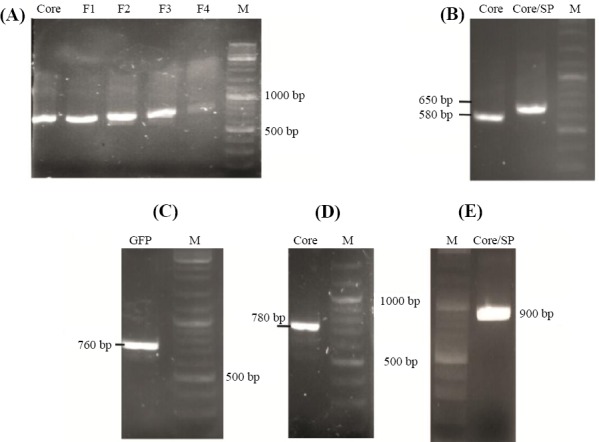
The result of amplification of coding sequences and splicing by overlap extension-PCR reaction analyzed by gel electrophoresis. (A(PCR products of the Core and SP-Core coding sequences, (B) PCR products related to the step-by-step addition of the SP to the Core-coding sequence using SOE-PCR. F1-F4 indicate the PCR result of using primers F1-F4 (Table 2) to extend core sequence. PCR result of cloning confirmation of (C) GFP, (D) Core, and (E) SP-Core in pGE-30446-HCE using the forward primer of the M13 and the reverse primer of the *core* gene. M, marker

In order to secrete the Core protein from the DCs, the sequence of the SP of the CD86 marker (accession number: P42081) on the surface of the DCs was added to the 5’ region of the *Core* gene using overlap extension PCR method (Fig. 2A). Four pairs of primers used for the amplification of the fragment are listed in Table 1. The amplified PCR product was digested by *PstI* restriction enzymes and was subcloned into the pGE-30446-HCE synthetic plasmid. To construct a reporter gene, the sequence of the green fluorescent protein (GFP) from pEGFP-N1 vector was amplified

(Table 1) and ligated into pGE-30446-HCE vector using the *Bst*BI restriction enzyme. The accuracy of the constructs was verified by sequencing.

**Table 1:** Gene specific-primers used to amplify the *core*, *SP*-*core*, and *GFP* genes.

Gene	Forward Primer (5’- 3’)	Reverse Primer (5’- 3’)
*Core*	ATATATCTGCAG**ATG**AGCACGAATCCTAA	ATATATCTGCAGTCAGGC**TGA**CGCGGGCACAGTCA
	F1:CTGCTGAGCGCC**ATG**AGCACGAATCCTAA	
	F2:GAGCAACATCCTGTTCGTGATGGCCTTCCTGCTG	
*SP- Core*	F3:AGTGCACCATGGGCCTGAGCAACATCCTGTTCACGCG	ATATATCTGCAGTCAGGC**TGA**CGCGGGCACAGTCA
	F4:ATATATATCTGCAGATGGACCCCCAGTGCACCATGG	
*GFP*	AGGAGTATTCGAACTATGGCGAGGAGC	ATATACTTCGAAACAGCTCGTCCATGCC

Start and stop codons are shown in bold.

### Preparation of *in vitro* transcription mRNA

IVT-mRNAs were synthesized from pGE-Core, pGE-SP-Core, and pGE-GFP vectors (Fig. 1B, 1C, and 1D) after the linearization of the constructs with *Hind*III and *Gsu*I restriction enzymes (MBI Fermentas, St Leon-Rot, Germany). The IVT-mRNAs synthesis was performed using RiboMAX™ Large-Scale RNA Production Systems-SP6 and T7 Kit (Promega Corporation, Madison, USA) according to the manufacturer’s instruction. DNA templates were purified using a PCR purification kit and used for *in vitro* transcription. Transcription of 5-10 µg of the DNA templates was carried out in a final 20–200 µl reaction mixture at 37 °C for 4 hours in order to generate uncapped IVT- mRNA. To remove the template DNA, 1 μl of RQ1 RNase-Free DNase (Catalog Numbers: M6101) was added to the IVT reaction mixture and incubated at 37°C for 15 min. Purification of mRNA was performed after DNaseI digestion by Total RNA Purification Kit (Jena-Bioscience , Germany), according to the instructions provided by manufacturer. The purity and quality of the RNAs were determined using spectrophotometric analysis and run on the agarose gel (1.1%). The mRNAs were stored in the RNase-free water at -80 °C.

### Computational analysis of mRNA secondary structure

The secondary structure of designed mRNA was analyzed using the RNA structure software package (http://rna.urmc.rochester.edu/RNAstructureWeb). The free energy of the mRNA was calculated using a dynamic planning algorithm to predict the most stable molecule. RNA structure software not only displays the thermodynamic properties of the sequence but also includes an interactive graphical representation of the predicted secondary structure[28].

### Generation of monocyte-derived DCs

Human monocyte-derived DCs were generated as described elsewhere[29]. Briefly, fresh human peripheral blood mononuclear cells were isolated from healthy donors by density gradient centrifugation over Ficoll-Hypaque. The blood cells were cultured for 2 h. Adherent monocytes were washed with RPMI-1640 medium and cultured for seven days in RPMI-1640 medium supplemented with 10% FBS, L-glutamine (2mM), penicillin (100 IU), streptomycin (100 µg/mL), IL-4 (25 ng/mL), and recombinant granulocyte-macrophage colony-stimulating factor (50 ng/mL) in a CO_2_ incubator at 37 °C. After seven days, immature monocyte-derived DCs (imoDCs) were ready for treatment.

### Transfection of imoDCs with IVT-mRNA

moDCs were transfected with 1.5 μg of IVT-mRNA using Lipofectamine 2000 (Invitrogen, USA), an RNA transfection reagent, according to the manufacturer’s instructions. The modification of IVT-mRNA with lipofectamine was performed as per manufacturer’s protocol. The moDCs were then incubated in a CO_2_ incubator at 37 °C for 18-48 h prior to testing for transgene expression. The expressions of GFP and HCV core protein were measured 24-48 h after transfection by fluorescence microscopy (Nikon Eclipse TE2000-U, USA) and Western blotting assay.

### Detection of Core and GFP protein expressions in DCs by Western blotting

After the transfection of imoDCs with IVT Core and GFP mRNA, the transfected and control cells were sonicated in a protein lysis buffer containing 50 mM of Tris, 50% glycerol, 0.1% Triton X-100, 1 mM of anti-protease (phenylmethylsulfonylfluoride). The acetone-precipitated proteins were loaded on 10% SDS-polyacrylamide gel and subsequent Western blot analysis was carried out using positive anti-HCV antibody serum specimens, which had been diluted 1:100 in 1× TBS buffer. After washing, the membrane was incubated with secondary peroxidase-conjugated anti-human IgG antibody for 2 h, followed by washing with TBST buffer. The bands were visualized using the chromogenic substrate 3,3’-diaminobenzidine (Sigma, USA). The untransfected cells were included as negative controls. The expression of GFP protein in the transfected cells was analyzed in the same way using 1:1500 diluted anti-GFP- antibody (Abcam, UK) and 1:5000 diluted anti-rabbit antibody conjugated with Alkaline phosphatase as the first and second antibodies, respectively. The protein was visualized by adding BCIP/NBT solution as a detection substrate.

## RESULTS

### Stable secondary structure of Core mRNA

**Fig. 3:**
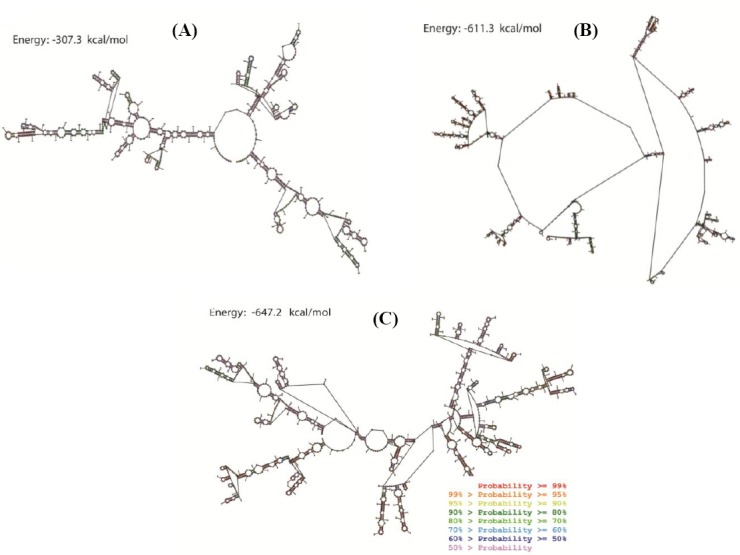
Predicted probability of nucleotides being paired or single stranded using RNA structure program. Predicted probability of nucleotides of (A) synthesized backbone sequence RNA, (B) Core protein RNA, and (C) SP-Core RNA. Probability lower than 50% is not colored. The calculated minimum free energies for secondary structure of synthesized sequence alone and containing Core and SP-Core coding sequences were -307.3, -611.3, -647.2 kcal/mol, respectively.

The structure of synthesized fragment containing 5’ UTR, T7 promoter, multiple cloning site, IRES, and 3’ UTR (915 bp) with and without Core (1497 bp) and SP-Core (1565 bp) proteins coding sequences was predicted using RNA structure program. RNA structure utilizes the nearest neighbor parameters and thermodynamic parameters to predict the lowest free energy structure of RNA[30]. In general, among a series of same-sized RNAs, lower thermodynamic energy represents a more stable structure. Figure 3 shows the predicted RNA structures with the lowest free energy for three designed RNAs. In the predicted structures of Core and SP-Core, several regions with paired and unpaired nucleotides have been indicated. Single-stranded regions indicate regions with the low probability of pairing. The calculated minimum free energy for the secondary structure of synthesized sequence alone and containing Core and SP-Core coding sequences were -307.3, -611.3, -647.2 kcal/mol, respectively. We compared the calculated free energy of HCV synthetic mRNAs with that of native mRNA sequences carried out in other studies with similar lengths of sequences, assuming that the synthesized mRNA in this study had a reasonable stability (Table 2)[31,32].

**Table 2:** Minimal free energies of native mRNA with predicted energies for mRNA synthesized in this study

Gene name	Native mRNA		Gene name	Synthetic mRNA	
	
	Length	MFE		Length	MFE
*HUMGST (glutathione S-transferase)*	909	-204	Synthetic sequence	915	-307.3
*THARGAA(tRNA)*	1471	-650.1	Synthetic sequence (with *Core* gene)	1497	-611.3
			Synthetic sequence (with *SP-Core* gene)	1565	-647.2

MFE, minimum free energy

### Preparation of template plasmid expressing pGE-Core, pGE-SP-Core, and pGE-GFP proteins

The sequences related to the 5’ UTR, T7 promoter, MCS, IRES, and 3’ UTRs of the plasmid with the length of 915 bp were synthesized by GeneRay Co. We assumed that adding known 5’ and 3’ UTRs to the construct will stabilize the IVT mRNA structure and will increase the efficiency of translation. Based on the studies conducted in this area, a structure of the 5’ UTR of human hsp70 enhances the translation of mRNA in mammalian cells and is predicted to be valuable in the context of genetic vaccination[33,34]. To confirm the subcloning of Core and SP-Core coding sequences in *PstI* restriction site, PCR test was conducted using specific primers (Fig. 2B). In order to confirm the subcloned GFP sequence by *Bst*BI restriction enzyme, the resulting clone was amplified using GFP-specific primers (Fig. 2C). GFP is a widely used reporter protein for monitoring gene expression *in vivo* and *in vitro* and for investigating the intracellular pattern of protein localization and trafficking. As GFP is more sensitive than other reporter genes, it requires no special cofactor for detection. Also, the intensity of GFP fluorescence is directly proportional to GFP mRNA abundance in the cells[35,36]. In the current study, GFP protein was used as a quantitative reporter of gene expression in individual DCs. Clones with the correct orientation of gene were selected with a confirmatory PCR reaction using the forward primer of the M13 and the reverse primer of the *Core* gene (Fig. 2D and 2E).

### *In vitro* transcription process

**Fig. 4:**
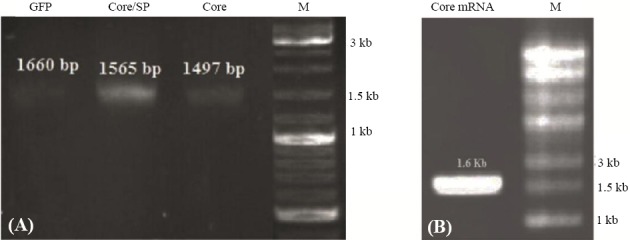
PCR products of cloned sequences and mRNA transcript. (A) The amplified fragments obtained from the enzymatic digestion of plasmids carrying *Core*, SP-*Core*, and *GFP genes*; (B) The amplified HCV *core* mRNA transcript (1.6 kb) running on an agarose gel (1.1%). M, RNA marker

To perform a T7 IVT, prepared plasmids containing DNA sequence with known flanking sequences were digested by *Hind*III and *Gsu*I restriction enzymes. The lengths of resulting fragments for plasmids containing *Core*, *SP-Core*, and *GFP* coding sequences were 1497, 1565, and 1660 bp, respectively. These fragments were purified, and the quality of the generated DNA was determined using spectrometry, calculating A260/A28 0 ratio, as well as agarose gel (1.5%) electrophoresis (Fig. 4A). *In vitro* transcription was performed from DNA templates. RNA concentration was assayed by a spectrophotometric analysis at OD 260 nm. The concentration of mRNA synthesized in a triplicate experiment was between 2-2.4 mg/mL of RNase-free water. The purity of the mRNA was determined by A260/A280 ratio, which was in the range of 1.6 to 2.0. Given the normal range of 1.8 to 2.1 for the ratio, the quality of synthesized RNAs was acceptable. To check the size and the quality of mRNA, a small sample of synthetic mRNA was loaded on a 1.1% agarose gel. After electrophoresis, the HCV Core mRNA transcript with a length of about 1.6 kb was detected on an agarose gel (Fig. 4B).

### Effective expression of designed mRNA in moDCs

**Fig. 5:**
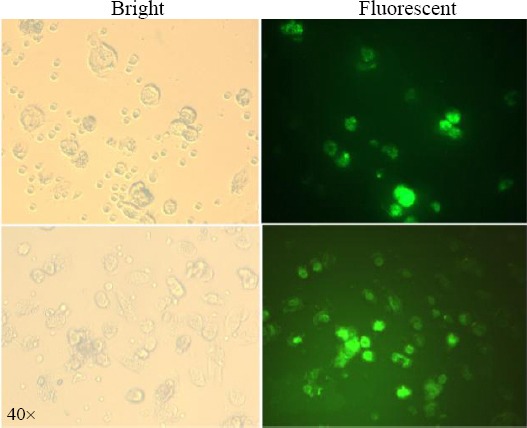
GFP expression in DCs using fluorescence microscopy. Immature DCs were transfected with IVT-mRNA expressing GFP and were analyzed for GFP protein expression 48 h after transfection. GFP protein expressed in DCs is indicated in fluorescent microscopy fields (right).

**Fig. 6:**
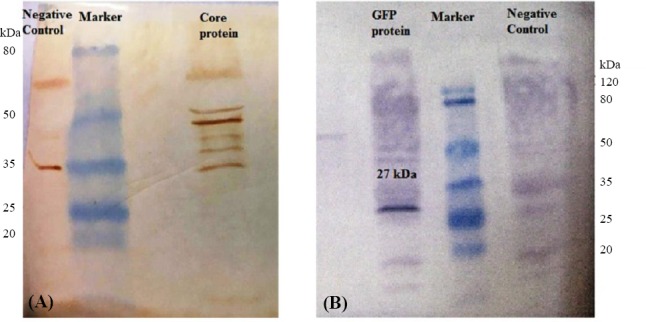
Detection of expressed Core and GFP proteins by Western blot assay. (A) Core protein in transfected DCs. The right line of the marker shows Core protein, as a multi-band protein, with different molecular masses (37-55 kDa) in the transfected DCs, and the left line of marker shows the protein extracted from the untransfected DCs, as the negative control; (B) GFP protein in transfected DCs. The right line of marker is protein extract from the untransfected DCs, as negative control, and the left line of marker is GFP protein with the expected molecular mass (27 kDa) in the DCs extract.

The transfection of moDCs using lipofectamine resulted in a relatively good transfection efficiency. The GFP expression in the cells was measured by fluorescence intensity and Western blotting. The moDCs were transfected using 2 μg of GFP mRNA along with 2 μl of Lipofectamine 2000. After 24-48 h, the GFP expression in the cells was investigated by fluorescence microscopy (Fig. 5). The analyses demonstrated strongly fluorescent moDCs. The Core protein expression was assayed in the moDCs via Western blotting. As illustrated in Figure 6A, Core protein was observed as a multi-band protein with different molecular masses (37-55 kDa) in the cell extracts obtained after the transfection of the moDCs. The observed additional bands, particularly the one with the molecular weight of 44 kDa, might correspond to the dimer form of the Core protein. The presence of proteins with other molecular weights suggests that the formation of complex products is possible. Also, the expression of GFP in moDCs was screened by Western blotting after the transfection of its mRNA with lipofectamine. As shown in Figure 6B, a protein with the expected molecular mass (27 kDa) was observed in the cell extracts. The Western blotting on untransfected moDCs, as negative control, has also been carried out (Fig. 6).

## DISCUSSION

In the field of vaccine development, it is necessary to pay attention to its scientific, technological, and economic aspects. Therefore, the challenges that need to be addressed are (A) the lack of efficiency against highly variable and/or persistent pathogens, (B) the development of a cheaper and more flexible technology to use in the absence of resources, and (C) better adaptation for individual or rapid production. Considering the mentioned issues and the characteristics of synthetic mRNA, it seems that synthetic mRNA could be of particular importance in the future of vaccine development, especially for highly variable infectious agents[37,38].

Synthetic mRNA allows creating the optimal immunogenic characteristics of a pathogen by eliminating unnecessary or harmful aspects of a pathogen. Due to its chemical and pharmacokinetic nature, IVT-mRNA may mimic the RNA of viruses; however, it causes an infection in a transient manner[39]. To design an effective mRNA-based vaccine, it is essential to select an appropriate antigen and design mRNA with the correct structure composition. A common vector designed for the mRNA-based vaccine comprises of an ORF encoding the antigen of interest and optimized cis-acting flanking structures, with the 5’ and 3’ UTRs adjacent to the ORF, the terminal 5’ 7-methylguanosine cap structure (cap), and the 3’ poly (A). All of these elements help to enhance the antigen yield via maximizing translation speed and/or vector stability in transformed cells[40].

Initiating a translation independent of the 5’ cap has been well understood for viral mRNAs. For the first time in poliovirus, an alternative mechanism was found to start the translation, which occurs independent of the 5’ cap[41]. Some viral mRNAs, such as picornaviral RNAs and HCV RNAs, often contain elements that directly promote translation initiation. One of these specific factors is a highly structured 5’ UTRs, known as IRES, that can recruit ribosomes to the internal sequences of the mRNA lacking of 5’ cap structure. This mechanism is often exploited by mRNA expressions from the genome of the virus-infecting eukaryotic cells[41,42]. Typically, in some eukaryotic cells, the translation of mRNAs begins with the recruitment of the 43S ribosomal complex to the 5’ cap of mRNAs. However, some transcriptions are translated independently of the 5’ cap through unknown mechanisms[43].

Meyer *et al*.[43] have defined a unique translation initiation mechanism that does not require the 5’ cap. Uncapped, luciferase-encoding mRNAs containing a modified β-globin 5’UTR with a single m^6^A residue were used for *in vitro* translation assays. The results showed that the m^6^A in the 5’ UTR can bind to the eukaryotic initiation factor 3. The analysis of the transcriptome profiles showed that the translation of mRNAs from 5’ UTR m^6^A decreases with the deletion of m^6^A methyl transferase[43]. Also, in the tobacco necrosis necrovirus, RNA is naturally translated, despite the lack of both 5’ caps and poly (A) effectively, that is partly due to the presence of a component in 3’ UTRs, which is called Barley yellow dwarf virus-like cap-independent translation element (BTE). Accordingly, in a study, the RNA encoding of luciferase reporter gene along with viral UTRs was used and uncovered a sequence downstream of the BTE that is required for translation without poly (A) *in vivo*[44]. Another study by Vivinus *et al*.[33] have examined the mechanisms regulating the translation system for cellular proteins such as heat shock proteins that continue to be synthesized, despite the risk of translation conditions. They found that the 5’ UTR of hsp70 mRNA contributes to the cap-independent translation without exhibiting typical features of IRES, but it does not behave as the viral IRES. In this study, 5’ UTR of mouse hsp70 was used[33]. The effective translation of several mRNAs with the involvement of IRES in a number of cellular conditions is maintained as the cap-dependent protein synthesis is reduced. For instance, IRESs of the vascular endothelial growth factor and hypoxia-inducible factor-1a genes increase the translation of the corresponding mRNAs in hypoxic cells[45].

The analyses of studies followed the translation process without 5’ cap and poly (A) *in vitro* have shown that the absence of 5’ cap and poly (A) compensates with the presence of IRES sequences in the upstream and downstream regions of the gene in the structure of the mRNA, and despite this defect, the translation process is done properly. Considering that most RNA viruses have the same structure and that such a structure is naturally responsive to the expression of viral proteins, it can be modeled to design and synthesize mRNA expressing variant antigens.

In this study, we considered the natural pattern of the RNA structure of the virus for the synthesis of the IVT-mRNA. Designed HCV RNA does not possess 5’ cap and poly (A) tail . Instead, the IRES element (340 nucleotides) is located close to the 5’ end of the viral genome[46]. The results of cell transfection demonstrated that despite the absence of the 5’ cap and poly(A) tail, the structure of the IVT-mRNA was stable and was able to express the genes that it carried. The 5’ cap plays an important role in the ribosomal recognition of messenger RNA when translated into a protein, and the poly(A) tail can also stimulate translation and cooperate with the cap structure[47]. It seems that the absence of the 5’ cap and poly (A) tail in this structure is offseted by the presence of the IRES sequences that can directly recruit ribosomes under stress conditions and can bypass the need for a 5’ cap, which is normally recognized by the translation initiation complex.

We designed a modified HCV antigen-coding mRNA, and computational evaluation showed its stability to create a stable secondary structure. Core antigen-coding sequence of HCV was successfully cloned into GE-30446-HCE vector, and its expression in the imoDCs transfected by transcribed IVT-mRNA confirmed the accuracy of designed mRNA. Considering the stability of the designed mRNA and its efficient expression in the effector cells such as moDCs, the synthesized IVT-mRNA can be used for further attempts in the development of RNA vaccine against HCV.
